# Phylogenetic supertree reveals detailed evolution of SARS-CoV-2

**DOI:** 10.1038/s41598-020-79484-8

**Published:** 2020-12-22

**Authors:** Tingting Li, Dongxia Liu, Yadi Yang, Jiali Guo, Yujie Feng, Xinmo Zhang, Shilong Cheng, Jie Feng

**Affiliations:** 1 Institute of Immunology, School of Basic Medical Sciences, Lanzhou University, Lanzhou, 730000 China; 2 Institute of Pathology, School of Basic Medical Sciences, Lanzhou University, Lanzhou, 730000 China; 3 The Second Clinical Medical School, Lanzhou University, Lanzhou, 730000 China; 4 The First Clinical Medical School, Lanzhou University, Lanzhou, 730000 China

**Keywords:** Virology, SARS-CoV-2, Viral epidemiology, Viral evolution, Systems virology

## Abstract

Corona Virus Disease 2019 (COVID-19) caused by the emerged coronavirus SARS-CoV-2 is spreading globally. The origin of SARS-Cov-2 and its evolutionary relationship is still ambiguous. Several reports attempted to figure out this critical issue by genome-based phylogenetic analysis, yet limited progress was obtained, principally owing to the disability of these methods to reasonably integrate phylogenetic information from all genes of SARS-CoV-2. Supertree method based on multiple trees can produce the overall reasonable phylogenetic tree. However, the supertree method has been barely used for phylogenetic analysis of viruses. Here we applied the matrix representation with parsimony (MRP) pseudo-sequence supertree analysis to study the origin and evolution of SARS-CoV-2. Compared with other phylogenetic analysis methods, the supertree method showed more resolution power for phylogenetic analysis of coronaviruses. In particular, the MRP pseudo-sequence supertree analysis firmly disputes bat coronavirus RaTG13 be the last common ancestor of SARS-CoV-2, which was implied by other phylogenetic tree analysis based on viral genome sequences. Furthermore, the discovery of evolution and mutation in SARS-CoV-2 was achieved by MRP pseudo-sequence supertree analysis. Taken together, the MRP pseudo-sequence supertree provided more information on the SARS-CoV-2 evolution inference relative to the normal phylogenetic tree based on full-length genomic sequences.

## Introduction

Severe acute respiratory syndrome coronavirus-2 (SARS-CoV-2), a novel coronavirus emerged in December 2019, causes an ongoing outbreak of Corona Virus Disease 2019 (COVID-19). COVID-19 has caused a global pandemic, and as of 15 July 2020, 13,323,530 cases of infections have been confirmed in more than 188 countries/regions, with 578,628 deaths^1^. Actually, in addition to COVID-19, coronaviruses of SARS-CoV and MERS-CoV that created epidemics in the past are well-known to cause severe disease in humans. Although the above mentioned three viruses are all identified as beta-coronaviruses by the full-length genomic sequence analysis, SARS-CoV-2 divergent from SARS-CoV and MERS-CoV, belongs to a distinct lineage on the phylogenic tree^2^. More and more genomes of SARS-CoV-2 isolates were sequenced all around the world. It creates an opportunity for precisely analyzing the phylogeny and evolution of SARS-CoV-2^2–6^. However, SARS-CoV-2 isolates displayed barely detected phylogenetic distance among each other in the phylogenetic tree. Thus, the detailed evolution of SARS-CoV-2 is still under the veil. Methods for phylogenetic tree construction are mostly based on a single gene in coronavirus genome or one artificial gene composed of the full-length genomic sequence^4,6,7^. Notably, critical limitations exist in these approaches. Gene selection is a major problem that phylogenetic methods based on a single gene need to tackle^8,9^. Different phylogenetic trees can be yielded based on different genes, with the results of phylogenetic relationships not always consistent but often conflict^9,10^. Additionally, the legitimacy of using the full-length SARS-CoV-2 genomic sequence for phylogenetic analysis is challenged on the grounds that the size of genes consisting of coronavirus genome varies in a large range. Plenty of phylogenetic information retained in small-size genes would be drowned out, making phylogenetic methods based on full-length SARS-CoV-2 genomic sequences phylogenic results poorly reliable. Remarkably, ORF1ab gene (21,290 bp) in SARS-CoV-2 genome comprises about 75% of the whole genome sequence, while the genes of key functional and structural proteins, including S (3822 bp), E (228 bp), M (668 bp), and N (1260 bp), take up less than 22%. Furthermore, using a single gene or full-length genome sequence for phylogenetic analysis requires orthologous genes in all taxa, which would limit the employment of species with large phylogenetic distance as outgroup. For example, at least five proteins of SARS-CoV-2 fail to establish orthology relationships with proteins in MERS-CoV, and the ORF8 of SARS-CoV-2 has no orthologous proteins in SARS-CoV. Therefore, such phylogenies can seriously mislead evolutionary events in between.

Supertree method, whose embryonic theory was described in the 1980s^11,12^, combines a set of source phylogenetic trees to produce one comprehensive phylogenetic tree reasonably that is called supertree^13^. The source phylogenetic trees employed for supertree construction can be consistent or inconsistent or partly overlapped based on different genes or phenotypes. Supertree method exhibits its technical superiority for phylogenetic analysis of creatures that are lack of compatible data for analysis using a single optimization criterion. It can use the full phylogenetic dataset that is available and combine data in various forms, including DNA or amino acid sequences, morphology, immunological distances, etc., to produce the overall finest supertree. Indeed, loss of information caused by using the source trees to re-construct supertree is inevitable, yet simulation studies proved that this trade-off is an affordable cost to be able to integrate all possible sources of phylogenetic information, at least for the matrix representation with parsimony (MRP) supertree method^14,15^.

Supertree method has been widely used for phylogenetic analysis of the creatures with a large size of genomes, including mammals^16^, birds^17^, palms^18^, ray-finned fishes^19^, shrimps^20^, etc. For organisms possessing relatively small size of genomes, such as prokaryotes, multiple approaches of genomic phylogenetic analysis have been adopted. In particular, supertree analysis brings new insights into prokaryotic evolution that wasn’t resolved by many other approaches, e.g. supertree successfully supports the monophyly of Proteobacteria that includes *Helicobacter pylori* and *Campylobacter jejunii*, but has rarely been found with other genomic tree methods^21^. Moreover, the phylogenetic network based on supertree method revealed a non-vertical evolution scenario during the evolutionary history of haloarchaea, which is achievable for few other phylogenetic approaches relied on a single gene or the full-length genome sequences^22^. However, supertree method is rarely used for phylogenetic analysis of viruses. In this study, supertree methods were employed for phylogenetic analysis of SARS-CoV-2, aiming to figure out the origin and evolution of SARS-CoV-2 through phylogenetic supertree analysis.

## Material and methods

### Dataset construction

The full-length genomic sequences and protein-coding sequences (CDSs) of 102 SARS-CoV-2, 5 SARS-CoV, 2 MERS-CoV, and 11 bat coronaviruses were downloaded from NCBI Severe acute respiratory syndrome coronavirus 2 data hub (https://www.ncbi.nlm.nih.gov/labs/virus/vssi/#/) and GenBank (http://www.ncbi.nlm.nih.gov/genbank/) (Supplementary Table 1). Among genomic sequences of SARS-CoV and bat coronaviruses, those showing high similarity with genomic sequences of SARS-CoV-2 were chosen. The integrity of sequences was checked, and the fragmented sequences were reconstructed. Finally, the datasets were constructed by labeling the sequences with the region of sampling and collection date.

### Construction of phylogenetic tree with full-length genomic sequences

The full-length genomic sequences of 120 coronaviruses were aligned using the L-INS-i method of MAFFT v7.310^23^. Aligned sequences were converted into phylip file format by Clustal W^24^. Maximum likelihood (ML) trees based on full-length genomic sequences were constructed and estimated by PhyML program version 3.0^25^ with 100 bootstraps resampling. The phylogenetic trees were visualized by FigTree v1.4.4 (http://tree.bio.ed.ac.uk/software/figtree/).

### Construction of phylogenetic supertrees

**Figure 1:**
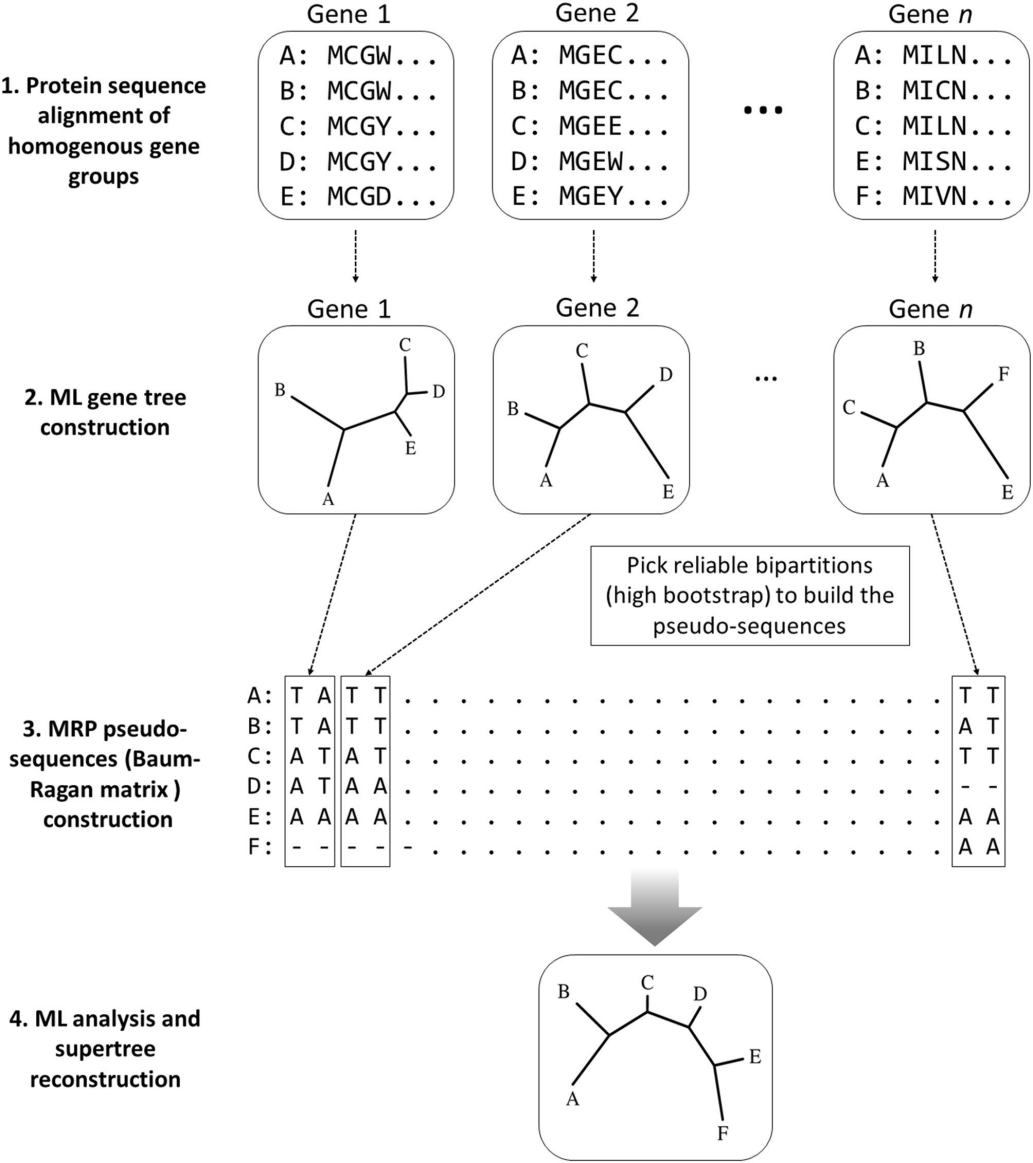
Schematic diagram of the MRP pseudo-sequence supertree method used in this study. Instead of using “0”, “1” and “?” to mark the bipartitions in the traditional MRP methods, pseudo-sequences supertree uses “T”, “A” and “–” to mark the bipartitions in the source tree. The pseudo-sequences were used for supertree reconstruction with well-established phylogenetic analysis.

The matrix representation with parsimony (MRP)^9,26^ pseudo-sequence supertree^22^ was built in this study. The construction steps of MRP pseudo-sequence supertree was briefly illustrated in Fig. 1. Firstly, ten groups of CDSs for orthologous proteins in selected coronaviruses were organized using the OrthoMCL program^27^, with repeated sequences removed from the orthologous groups. The CDSs of 120 coronaviruses were assigned to their corresponding orthologous protein groups by custom-made scripts, and aligned by MAFFT^23^ with the L-INS-i method, followed with formation into phylip file by Clustal W^24^. Secondly, ML phylogenies by using PhyML^25^ were employed to build source phylogenetic trees based on each CDSs, with 100 bootstrap replications. Thirdly, the members of each clade making up the selected bipartitions (above 55% bootstrap support) are assigned an A or T, and custom-made scripts were applied to retrieve the Baum-Ragan matrix pseudo-sequences as reported in our previous study^22^. Fourthly, The pseudo-sequences of the coronaviruses were used to re-construct the phylogenetic supertree using PhyML^25^. The A/T substitutions were treated equally in this analysis, without systematic bias imported.

In addition, published supertree software Clann (version 4.2.4) was also used to construct traditional MRP supertree (with PAUP* version 4.0a166^28^) and MSSA (most similar supertree method) supertree, with default parameter settings^29^. L.U.St package version 2.0^30^ was used to construct an approximated maximum likelihood supertree.

### Simulation-based method to evaluate the validity of MRP supertree on analysis of viral genomic evolution

Artificial Life Framework v1.0 (ALF)^31^ was used to simulate viral genomic evolution, taking trimmed bat coronavirus KJ473816 genomic sequence as the root. The trimmed genomic sequences composed of ten genes were adapted to shorten the phylogenetic analysis time. The simulated genomic evolution process proceeded at the setup of CPAM and TN93 substitution model^31^. Lateral gene transfer (LGT) setting was applied, due to its occurrence in the real evolution of RNA viruses^32^. Mutation rates among the 10 genes were variable and the corresponding parameters were setup respectively, as reported by the latest SARS-CoV-2 study^33^. After simulated evolution, full-length genome ML tree, MRP pseudo-sequence supertree, and traditional MRP supertree were constructed, and then they were compared with the real tree generated by ALF.

### Mutation analysis of the SARS-CoV-2 clades in the supertree

Amino acid sequences of the viral genes were aligned by MAFFT^23^ and displayed in MEGA X^34^. Mutation sites on sequences of SARS-CoV-2 positioned in subclades in the phylogenetic supertree were identified manually.

## Results and discussion

### Comparison of MRP pseudo-sequence supertree and ML tree

**Figure 2:**
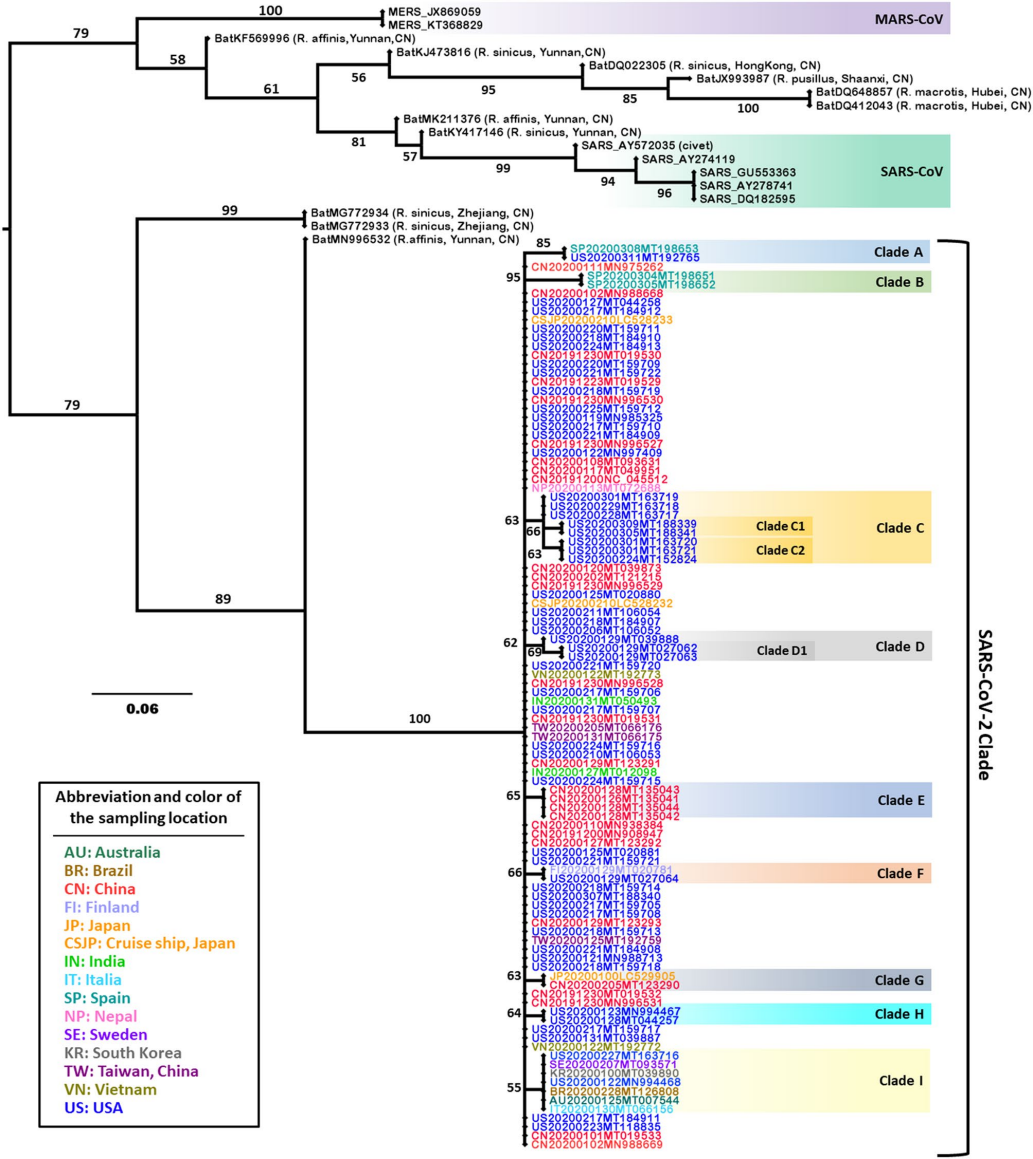
MRP pseudo-sequence supertree for SARS-CoV-2 constructed from protein source trees. The hosts and sampling locations of animal coronaviruses are enclosed in parentheses. The coding of SARS-CoV-2 viruses is the combination of the abbreviation of sampling location, sampling time, and Genbank accession. MERS-CoV clade, SARS-CoV clade, and nine clades of SARS-CoV-2 are highlighted and labeled, respectively. The numbers along the branches mark the bootstrap values percentage out of 1000 bootstrap resamplings.

**Figure 3:**
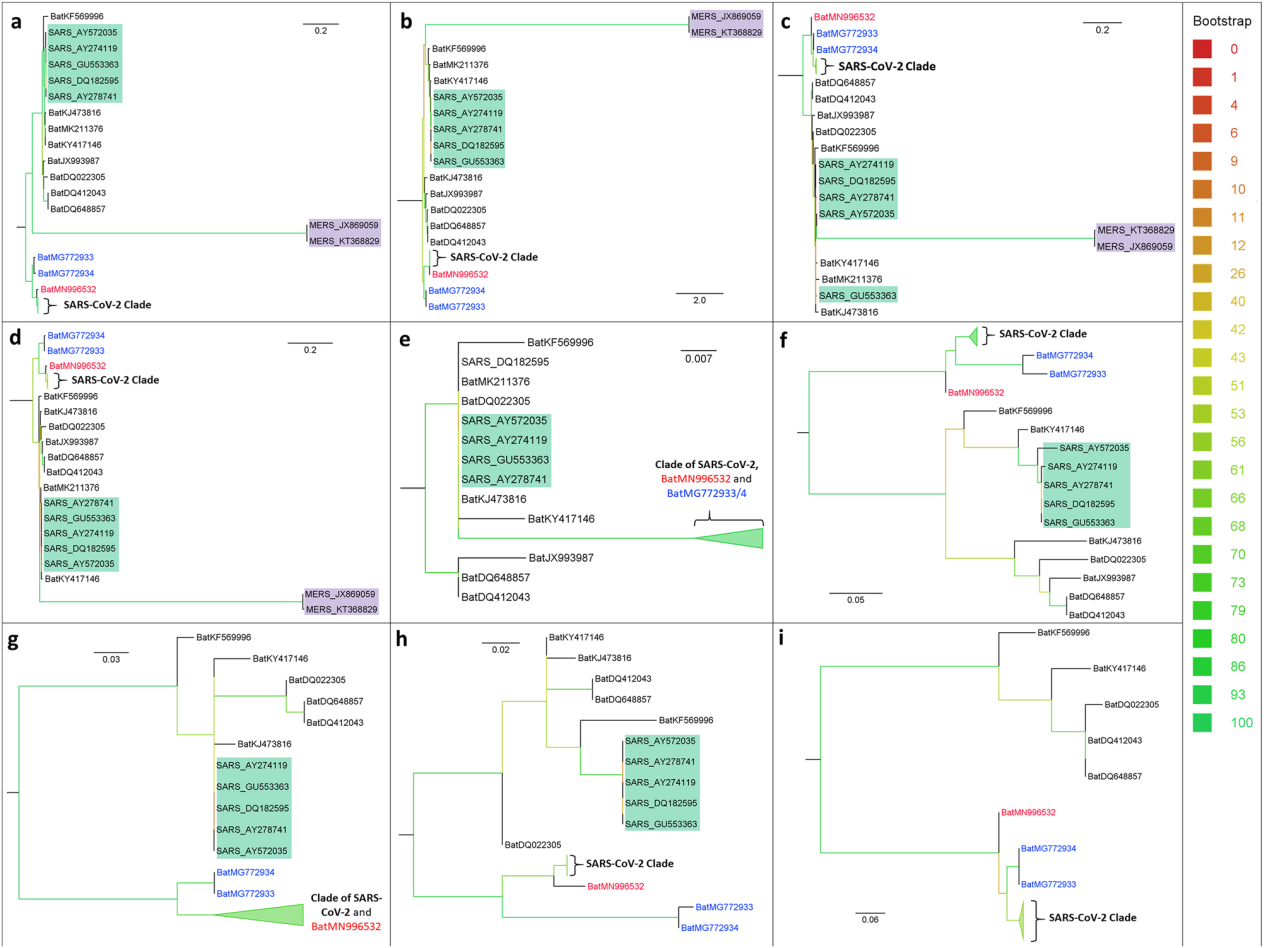
Source phylogenetic ML trees for phylogenetic supertree construction. (**a**) ORF1ab; (**b**) Spike protein; (**c**) M protein; (**d**) N protein; (**e**) E protein; (**f**) ORF3a; (**g**) ORF6; (**h**) ORF7a; (**i**) ORF8. Clades of SARS-CoV-2 are in bold in all source phylogenetic ML trees. Bat virus MG996532 is written in red, MG772933 and MG772934 are in blue. Clades of SARS-CoV and MERS-CoV are highlighted in green and purple, respectively.

To accurately determine the evolutionary relationships among SARS-CoV-2, approaches of MRP pseudo-sequence supertree and ML tree were employed for phylogenetic analysis of 102 SARS-CoV-2 isolated all over the world together with 5 SARS-CoV, 2 MERS-CoV, and 11 bat coronaviruses as outgroups. In the MRP pseudo-sequence supertree (Fig. 2), SARS-CoV and MERS-CoV were placed on one major branch, while SARS-CoV-2 belonged to another major branch. The divergent location of SARS-CoV-2 relative to SARS-CoV and MERS-CoV on the MRP pseudo-sequence supertree was consistent with the results from the phylogenetic ML tree in this study (Supplementary Fig. S1). It was also supported by previous reports about the phylogeny of SARS-CoV-2 constructed with the whole genome^3,4,6^. However, some discrepancies present between the MRP pseudo-sequence supertree and the ML tree. In particular, the MRP pseudo-sequence supertree analysis showed more resolution power than ML tree approach. Distinctive phylogenetic distances on clades of SARS-CoV and SARS-CoV-2 in MRP pseudo-sequence supertree, explicitly presented evolutionary relationships among coronaviruses. Also, the MRP pseudo-sequence supertree successfully identified civet-sampled coronavirus AY572035 to be the closest ancestor of the SARS-CoVs (Fig. 2), which was highly consistent with the previous study^35^. What is more, the MRP pseudo-sequence supertree showed detailed evolutionary relationship of SARS-CoV-2, with nine sub-branches identified from Clade A to Clade I in Fig. 2. The reliability of phylogenetic inference of SARS-CoV-2 in supertree is sufficiently guaranteed by high bootstrap values between 55 and 95. Conversely, coronaviruses clustered tightly on clades of SARS-CoV and SARS-CoV-2 in phylogenetic ML tree (Supplementary Fig. S1), with barely discerned branch length (less than 0.001). It is worth noting that some bat coronaviruses sampled from the same animal host or/and same sampling location, displayed closer genetic distance in MRP pseudo-sequence supertree, which is rational and logical from the perspective of evolutionary progress. However, bat coronaviruses showed no definitive evolutionary relationship in the phylogenetic ML tree. The major factor that determines the topology of the phylogenetic ML tree appears to be the ORF1ab gene that is about 75% of the genome. It is readily explained by the fact that evolutionary relationship was similar in the phylogenetic ML tree relative to the source phylogenetic ML tree based on the sequence of ORF1ab gene (Supplementary Fig. S1, Fig. 3a). Taken together, the phylogenetic supertree displayed significant superiority for deciphering evolutionary relationships among coronaviruses.

### Comparison of different supertrees of coronaviruses

Since the birth of supertree theory, many methods have been developed for constructing supertrees from source trees, including MRP method^9,26^, most similar supertree algorithm (MSSA) method^36^, average consensus^37^, and newly developed approximated maximum likelihood (ML) supertree method^30^. Among them, the MRP method is the most widely used supertree method, based on which MRP pseudo-sequence supertree was derived. However, few of them have been used for constructing supertrees of viruses.

In this study, the above-listed approaches for supertree construction were all adopted, attempting to seek out which supertree approach is the best one to clarify the phylogeny of coronaviruses. The outcome that the SARS-CoV clade is located in the SARS-CoV-2 clade in supertrees built by approaches of MSSA supertree (Supplementary Fig. S2) and average consensus supertree (Supplementary Fig. S3), strongly demonstrated that these two approaches can’t provide reliable phylogenetic signal of coronaviruses. Similarly, the ML supertree method is also improper for phylogenetic reconstruction by virtue of failure in resolution for the outgroup MERS-CoVs (Supplementary Fig. S4). Conversely, supertrees obtained based on the traditional MRP method and MRP pseudo-sequence supertree method showed similar topology (Supplementary Fig. S5, Fig. 2), providing a good separation among MERS-CoV, SARS-CoV, and SARS-CoV-2. The MRP pseudo-sequence method is relatively more suitable for phylogenetic reconstruction, as many taxa with the same sampling position and time are accurately resolved in the same clade (Clade B, C, D, E and H). The rationality of using the MRP pseudo-sequence supertree method for phylogenetic analysis should partly ascribe to the removal of most unreliable bipartitions with low bootstrap values (< 55) during the reconstruction process. The preservation of unreliable bipartitions resulted in the MRP supertree with a chaotic topology, especially in SARS-CoV-2 clade (Supplementary Fig. S5). Moreover, the MRP pseudo-sequence supertree method can choose various well-established phylogenetic algorithms to calculate the branch length and bootstrap statistical test from the MRP pseudo-sequences, rendering itself an extra opportunity for accurately constructing phylogenetic supertree.

In addition, MRP pseudo-sequence supertree relied on nucleic acid source trees was also constructed (Supplementary Fig. S6) in this study, which inappropriately placed MERS-CoVs in the SARS-CoV-2 clade. The problem of nucleic acid source tree-based supertree could be caused by the fact that coronaviruses recombine frequently^38^ and some recombination breakpoints may misdirect the reconstruction of the supertree. In contrast, this problem could be avoided by constructing a supertree based on protein sequence (Fig. 2), which would exclude the breakpoints in non-coding regions and minimize the influence of nonsense and silent mutation in coding regions. Consequently, protein-sequence based MRP pseudo-sequence supertree is more reliable and accurate.

### Evaluate the validity of MRP supertree on analyzing simulation-based viral genomic evolution

To prove MRP pseudo-sequence supertree is more preferable for analysis of coronavirus phylogenetics, we used ALF simulation frame to compare MRP supertree with full-length genomic sequence ML tree. In comparison with the real tree generated by ALF (Supplementary Fig. S7a), both MRP supertrees could correctly resolve the topology of the phylogenetic tree, yet the MRP pseudo-sequence supertree (Supplementary Fig. S7c) showed more reasonable branch length relative to the MRP supertree constructed by Clann (Supplementary Fig. S7d). Of particular interest was that the taxon SE008 was placed on an inappropriate position—an inconsistent node in the ML tree (Supplementary Fig. S7b). The poverty of the ML method applied here principally could attribute to the LGT events introduced in the simulation, which could be firmly supported by the fact that the ML method constructed a phylogenetic tree fitting well with the corresponding real tree generated by ALF as long as no LGT in the simulation (data not shown). It has been well known that virus evolution is a complex interaction between viruses and hosts, in which RNA viruses exhibit remarkable genomic flexibility. Factors affecting viral genomic flexibility include, but are not limited by, LGT among viruses and hosts, recombination, gain, and loss of genes^32^. Therefore, viral evolution is so intricate that the current model was incompetent to precisely run the simulation. Primarily, LGT event in the evolution of SARS-CoV-2 cannot be ignored in the simulation process. At this point, the MRP supertree established its superiority compared with the full-length genomic sequence ML tree.

### Clues to the origin of the SARS-CoV-2

As the phylogenetic MRP pseudo-sequence supertree and ML tree exhibited, RaTG13 (MN996532), bat-SL-CoVZC45 (MG772933), bat-SL-CoVZXC21 (MG772934) and SARS-CoV-2s formed one major clade (Fig. 2, Supplementary Fig. S1). In particular, RaTG13 isolated from bat *Rhinolophus affinis* (Yunnan, China), is the closest relative of SARS-CoV-2s, which substantiates the previously reported phylogeny of SARS-CoV-2s constructed with the whole genome^39,40^. However, the phylogenetic distance of SARS-CoV-2s and RaTG13 was distinctly exhibited in the MRP pseudo-sequence supertree (Fig. 2); by contrast, it was barely observed in the phylogenetic ML tree constructed in this study (Supplementary Fig. S1) or previous report^39^.

To interpret the disparate proximity between SARS-CoV-2s and RaTG13 in MRP pseudo-sequence supertree relative to ML tree, we examined and evaluated the 10 source ML trees (Fig. 3), based on which the MRP pseudo-sequence supertree was built. Consistent with the results of MRP pseudo-sequence supertree and ML tree, RaTG13 (MN996532) is identified as adjacent coronavirus to SARS-CoV-2s in source ML trees based on phylogenetic analysis of five CDSs, including ORF1ab, spike protein, N protein, ORF6 and ORF7a (Fig. 3a, b, d, g, h). By contrast, bat coronavirus MG772933 and MG772934, both of which are isolated from bat *Rhinolophus sinicus* (Zhejiang, China)^41^, were the nearest relatives of SARS-CoV-2 s in source ML trees based on M protein, ORF3a, and ORF8 (Fig. 3c, f, i). In addition, phylogenetic analysis of E protein sequence showed that SARS-CoV-2s, MN996532, MG772933, and MG772934 are pinpointed on the same branch (Fig. 3e). The inconsistent phylogenetic relationship relied on diverse genes seriously casts doubt on the reliability of single-gene based phylogenetic analysis.

Whatsoever, the above distinct phylogenetic analysis results showed beyond a reasonable doubt that the rates of evolution on sequences of varied proteins in SARS-CoV-2s are highly non-uniform. There probably exists another bat coronavirus in divergent species as the adjacent ancestor of SARS-CoV-2, and/or SARS-CoV-2s already made advanced evolution in its animal host. Anyway, what is clear is that the actual validity of RaTG13 be the direct ancestor of SARS-CoV-2 is seriously questioned, although they share 96.5% identical genome sequence. Taking RaTG13 as the last common ancestor of SARS-CoV-2 would seriously mislead phylogenetic inference of SARS-CoV-2.

### Mutants and evolution of SARS-CoV-2

Within phylogenetic MRP pseudo-sequence supertree, nine sub-branches were resolved in SARS-CoV-2 clades, labeled from clade A until clade I in Fig. 2, which were absent in phylogenetic ML tree based on full-length genomic sequence analysis (Supplementary Fig. S1). The sub-branches displayed an evolutionary scenario of the SARS-CoV-2s in human hosts from December 2019 to March 2020 all around the world, at least based on 102 SARS-CoV-2 isolates in this study. By interrogating ten CDSs of SARS-CoV-2s, diverse mutations are disseminated within five viral proteins, which are ORF1ab, N protein, spike protein, ORF3a, and ORF8 (Table 1). Within most mutation sites described in this study, the original amino acid was substituted by another one possessing altered chemical properties, except L1599F in ORF1ab (clade A), V62L in ORF8 (clade H), and I1606V in ORF1ab (clade D1). Most strikingly, SARS-CoV-2s from the USA displayed common mutation in clades of A, C, D, F, H, and I, covering a large number of countries listed in this study, including Spain, Finland, Sweden, Italy, Brazil, Australia, and South Korea. In particular, detection of the identical mutation in ORF3a protein (G251V) in clade I indicated the spread of the G251V mutant happened at least in January 2020 or earlier, in Sweden, Italy, Brazil, Australia, and the USA.

**Table 1:** Common mutations sites of the sub-clades in the SARS-CoV-2 phylogenetic supertree.

Clade^a^		Genebank ID	Sampling location	Sampling date	Mutation sites	
A		MT198653	Spain	2020.03.08	ORF1ab: P4715L; Spike protein: D614G	
		MT192765	USA	2020.03.11		
B		MT198652	Spain	2020.03.05	ORF1ab: F3071Y; N protein: S197L; ORF3a: G196V; Orf8: L84S	
		MT198651	Spain	2020.03.04		
C		MT163717	USA	2020.02.28		ORF1ab: P5825L, Y5865C; Orf8: L84S
		MT163718	USA	2020.02.29		
		MT163719	USA	2020.03.01		
	C1	MT188339	USA	2020.03.09	ORF1ab: S7042F	
		MT188341	USA	2020.03.09		
	C2	MT152824	USA	2020.02.24	ORF1ab: T1840I	
		MT163721	USA	2020.03.01		
		MT163720	USA	2020.03.01		
D		MT039888	USA	2020.01.29		N protein: S194L
	D1	MT027063	USA	2020.01.29	ORF1ab: A117T, I1606V	
		MT027062	USA	2020.01.29		
E		MT135041	China	2020.01.26	ORF1ab: L1599F; Orf8: L84S	
		MT135044	China	2020.01.28		
		MT135042	China	2020.01.28		
		MT135043	China	2020.01.28		
F		MT027064	USA	2020.01.29	Spike protein: H49Y	
		MT020781	Finland	2020.01.29		
G		LC529905	Japan	2020.01	N protein: P344S	
		MT123290	China	2020.02.05		
H		MN994467	USA	2020.01.23	Orf8: V62L, L84S	
		MT044257	USA	2020.01.28		
I		MT093571	Sweden	2020.02.07	ORF3a: G251V	
		MT066156	Italy	2020.01.30		
		MT126808	Brazil	2020.02.28		
		MT007544	Australia	2020.01.25		
		MN994468	USA	2020.01.22		
		MT039890	South Korea	2020.01		
		MT163716	USA	2020.02.27		

^a^The clades are coded as in Fig. 2.

The ORF1ab gene, taking up 75% of the whole genome size of coronavirus, encodes a series of non-structural proteins (nsp), which assemble to facilitate viral replication and transcription. Mutations in amino acid sequence of ORF1ab present in most clades, including clades A, B, C, D1 in D, and E, which are involved in SARS-CoV-2s from Spain, the USA, China, but no identical mutation site was detected. Among them, a mutation from proline to leucine (P4715L) in ORF1ab, was located on Nsp12. To be noticed, Nsp12 is considered as a primary target for nucleotide analog antiviral inhibitors such as remdesivir. Thus, the mutation (P4715L) would possibly make anti-coronavirus treatment less effective^42,43^.

Spike protein, responsible for viral entry into host cells, exhibited two mutated sites distributed in clade A (D614G) and F (H49Y), respectively. The mutation site D614G in spike protein is located between receptor-binding domain (451–509) and polybasic cleavage site (682–685)^44^, which possibly can regulate the capability of SARS-CoV-2s binding to human host ACE2 receptor or involved in other steps related to the invasion of host cells. Further studies and clinical observations are needed to figure out whether mutation sites on various proteins could change the viral ability for infection and its pathogenicity.

## Conclusion

The supertree method is a powerful approach applied in the phylogenetic analysis of coronavirus. The distinct phylogenetic distance in the SARS-CoV-2 clade only can be detected by MRP pseudo-sequence supertree. Relied on this approach, our study rationally questioned the reliability of RaTG13 be the last common ancestor of SARS-CoV-2s, and revealed various common mutations in SARS-CoV-2s. Timely monitoring of the variation and evolution of SARS-CoV-2s would be favorable to treatment and control of COVID-19 and prevent its future outbreak.

## Supplementary information

Supplementary Table 1.

Supplementary Figures.

## Data Availability

The datasets analyzed during the current study are available in the GenBank repository, https://www.ncbi.nlm.nih.gov/genbank/.
