# Supplementary material for: Phylogenetic supertree reveals detailed evolution of SARS-CoV-2
Source: Sci Rep. 2020 Dec 22;10:22366. doi: 10.1038/s41598-020-79484-8 (PMC7755913; doi:10.1038/s41598-020-79484-8)
Supplement: Supplementary file 2 — Supplementary Figures. [file 41598_2020_79484_MOESM2_ESM.docx]

**Supplementary material**

**Phylogenetic supertree reveals detailed evolution of SARS-CoV-2**

Tingting Li^1^, Dongxia Liu^2^, Yadi Yang^1^, Jiali Guo^3^, Yujie Feng^3^, Xinmo Zhang^3^, Shilong Cheng^4^, Jie Feng^2*^

^1^Institute of Immunology, School of Basic Medical Sciences, Lanzhou University, Lanzhou, China

^2^Institute of Pathology, School of Basic Medical Sciences, Lanzhou University, Lanzhou, China

^3^The Second Clinical Medical School, Lanzhou University, Lanzhou, China

^4^The First Clinical Medical School, Lanzhou University, Lanzhou, China

*Corresponding author:

Jie Feng, PhD

Institute of Pathology

School of Basic Medical Sciences

Lanzhou University

Lanzhou, China

Email: jfeng@lzu.edu.cn


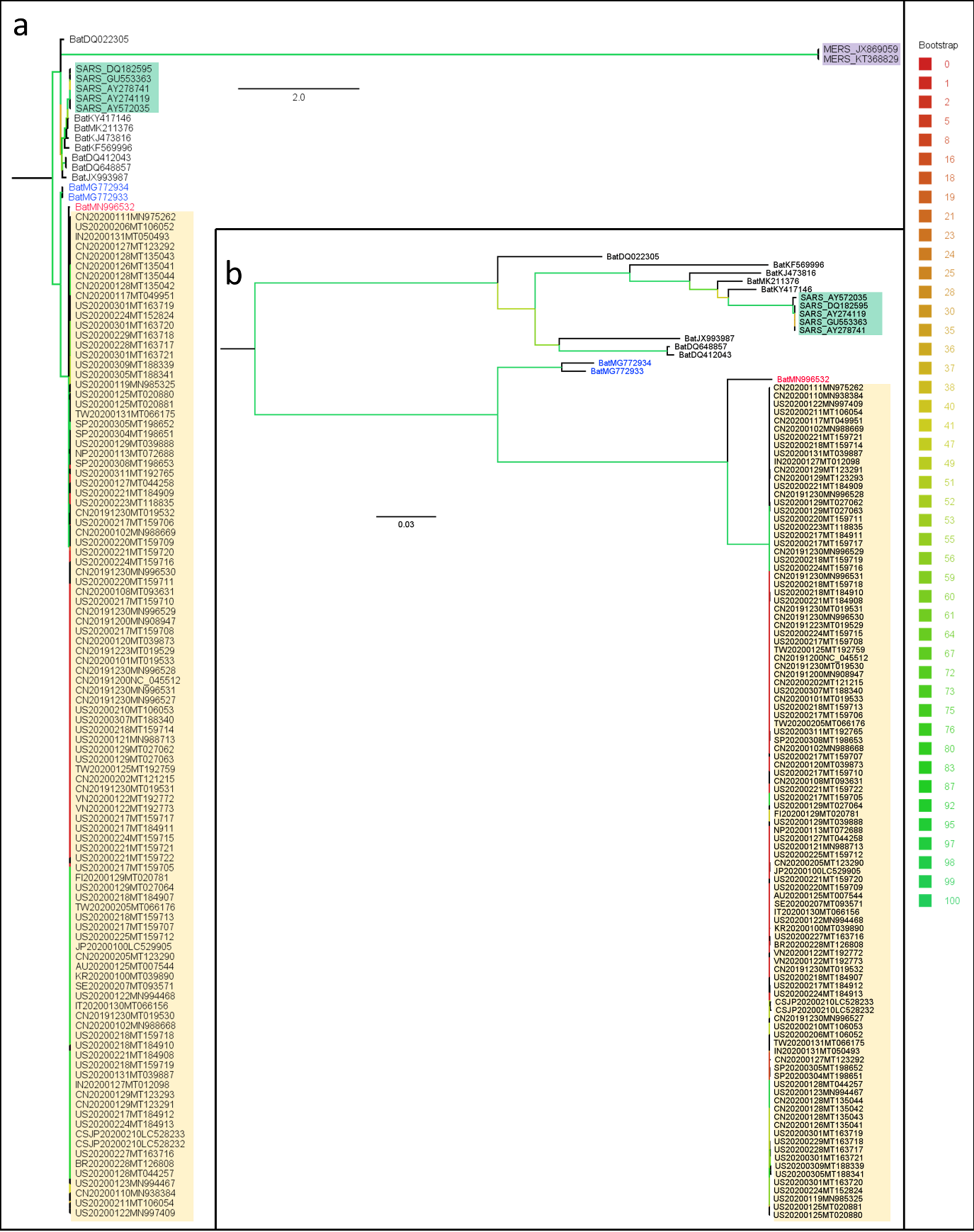


**Supplementary Figure S1.** The phylogenetic maximum likelihood (ML) tree of coronaviruses based on full-length genomic sequences. (a) The ML tree of 120 coronaviruses; (b) The ML tree of 118 coronaviruses, without MERS-CoV as outgroup. Bat virus RaTG13 (MN996532) is written in red, CoVZC45 (MG772933) and CoVZC21 (MG772934) are in blue. Clades of SARS-CoV-2, SARS-CoV and MERS-CoV are highlighted in yellow, green, and purple, respectively.


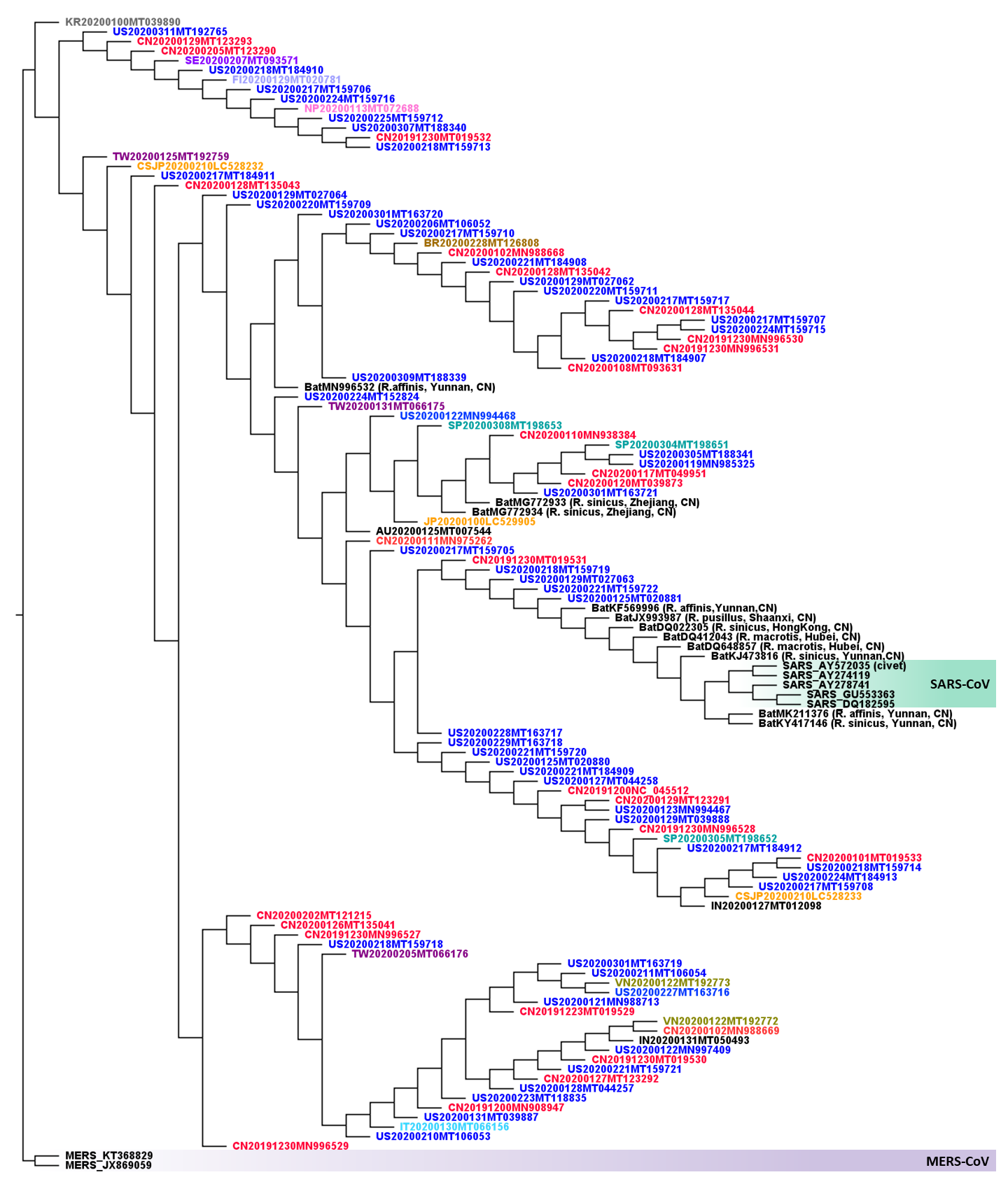


**Supplementary Figure S2.** The MSSA (most similar supertree method) supertree of coronaviruses re-constructed with the heuristic search of source trees using the dfit criteria implemented in Clann version 4.2.4.


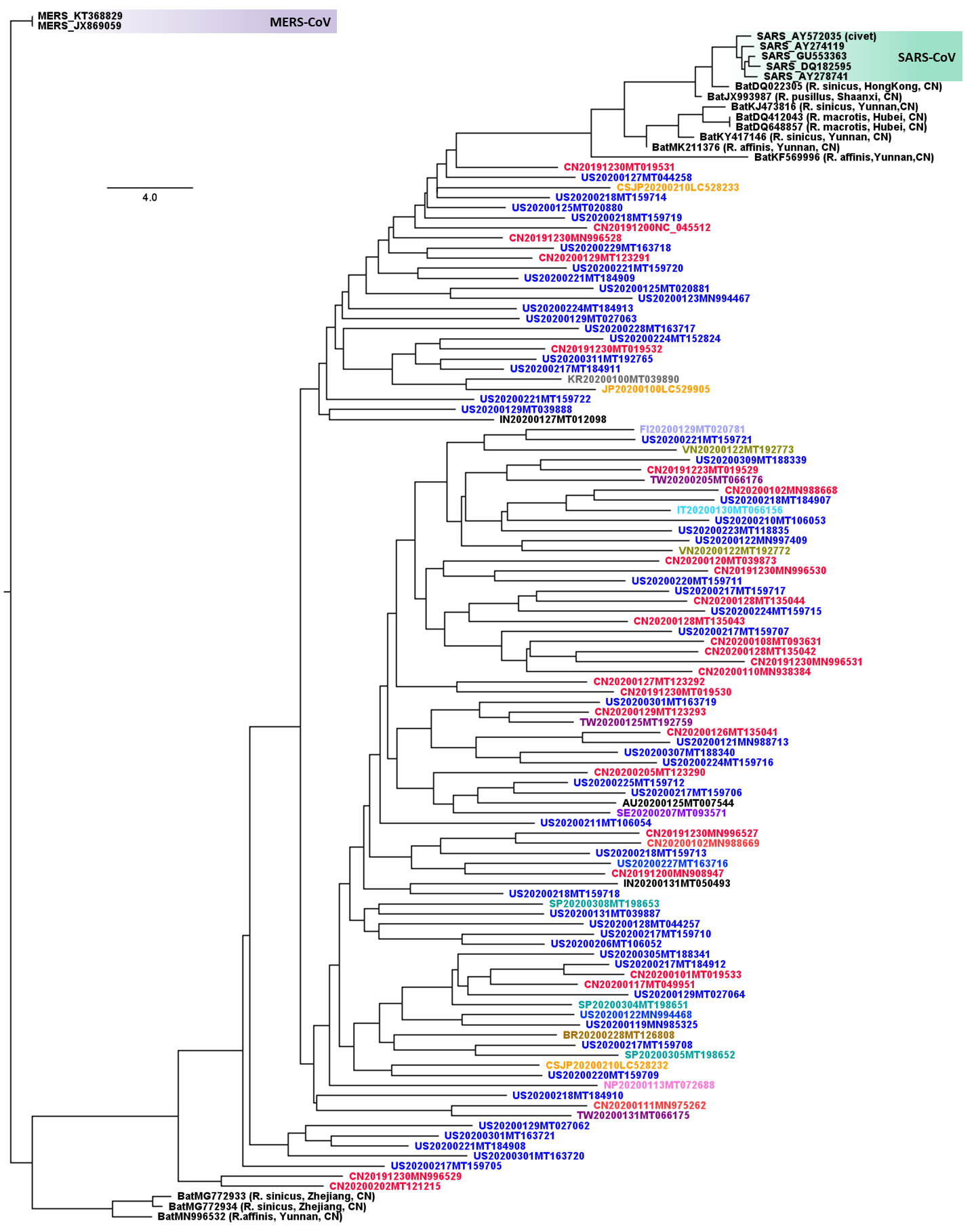


**Supplementary Figure S3.** The average consensus supertree of coronaviruses re-constructed with the avcon criteria implemented in Clann version 4.2.4, combined with the distance matrix analysis using PAUP* version 4.0a.


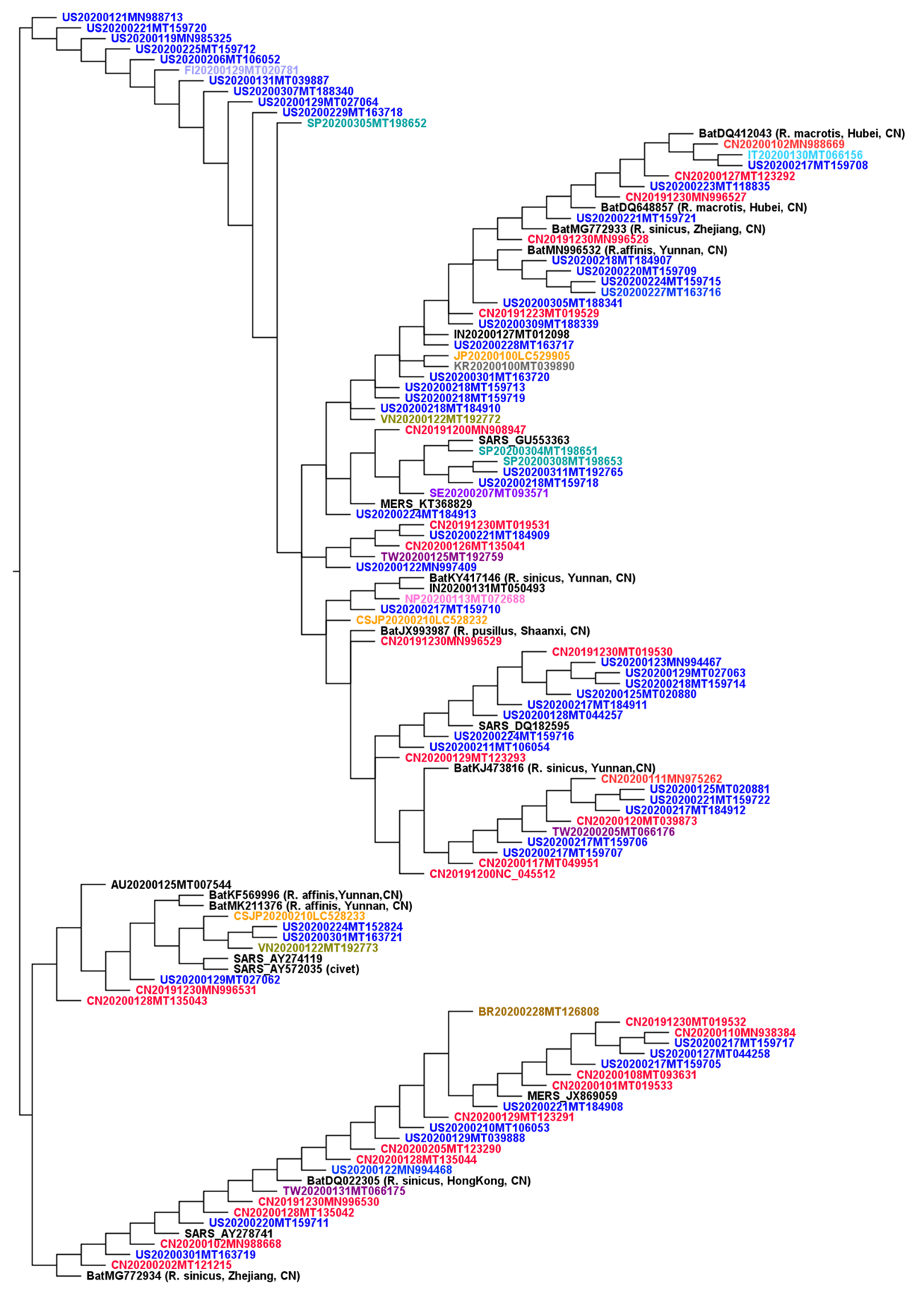


**Supplementary Figure S4.** The ML (maximum likelihood) supertree of coronaviruses re-constructed with L.U.St version 2.0.


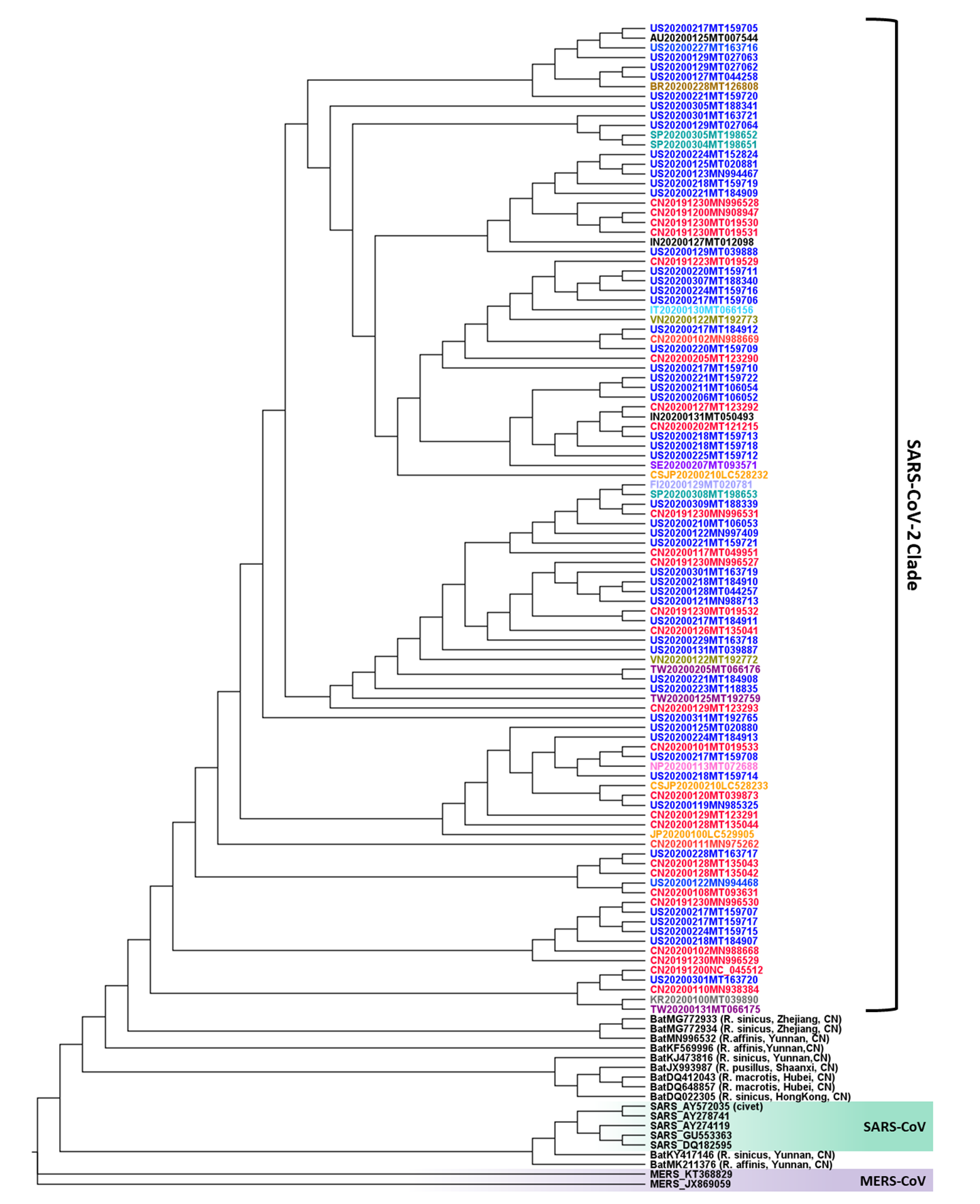


**Supplementary Figure S5.** The MRP (matrix representation using parsimony) supertree of coronaviruses re-constructed with the heuristic search of source trees using the MRP criteria implemented in Clann version 4.2.4, combined with the data matrices analysis using PAUP* version 4.0a.


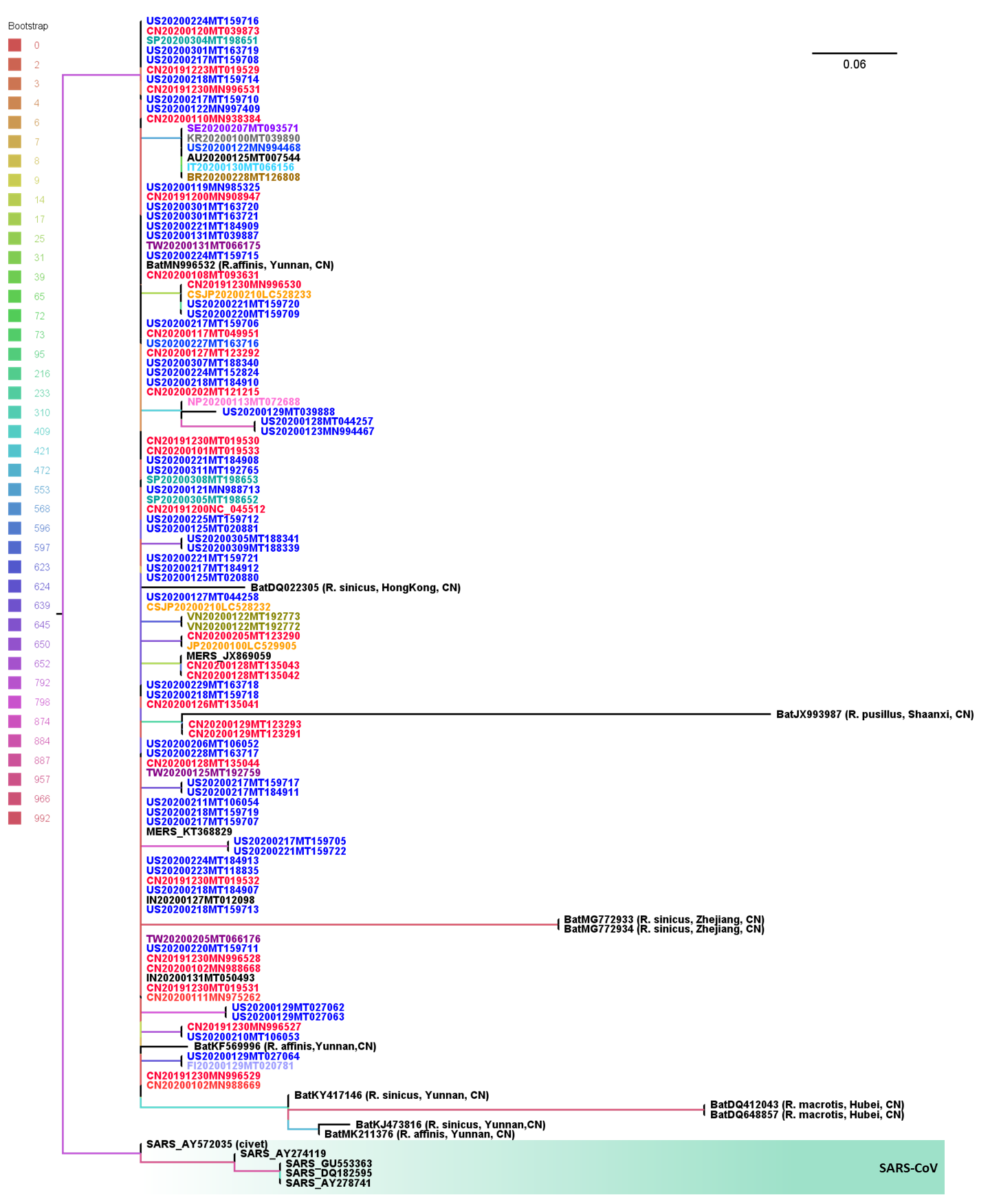


**Supplementary Figure S6.** MRP pseudo-sequence supertree for the SARS-CoV-2s constructed from nucleic acid source trees. The hosts and sampling locations of animal coronaviruses are enclosed in parentheses. The coding of SARS-CoV-2 viruses is the combination of the abbreviation of sampling location, sampling time, and Genbank accession. MERS-CoV clade, SARS-CoV clade, and nine clades of SARS-CoV-2 are highlighted and labeled, respectively. The numbers along the branches mark the bootstrap values out of 1000 bootstrap resamplings.


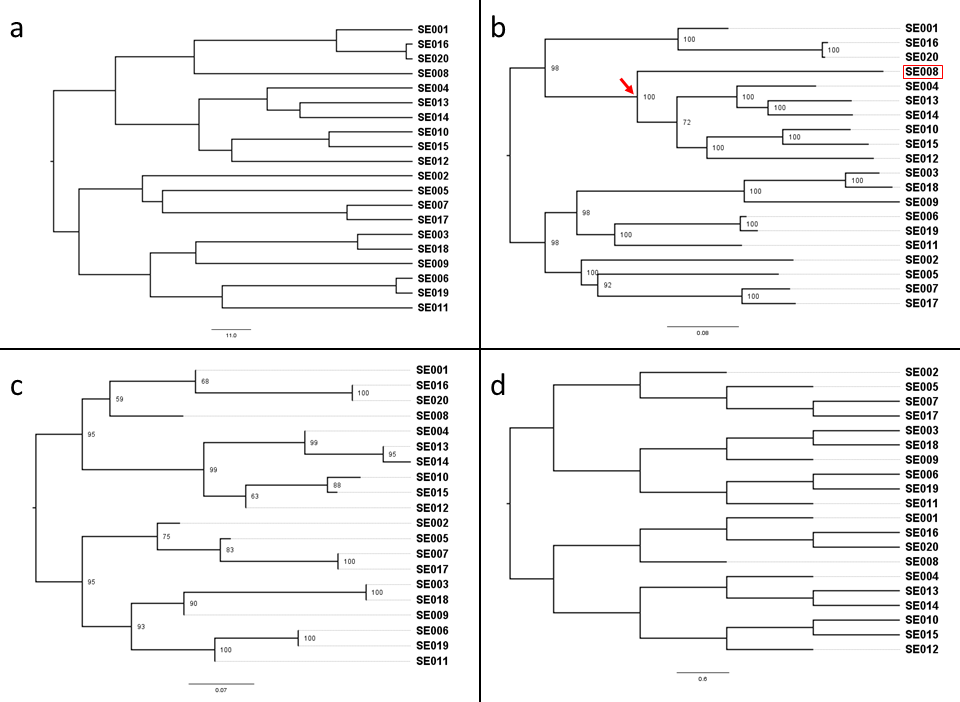


**Supplementary Figure S7.** Simulation evolution to evaluate the accuracy/uncertainty of MRP supertree and ML tree. a. the real tree generated by Artificial Life Framework v1.0 (ALF); b. the ML full-length genomic sequence ML tree; c. the MRP pseudo-sequences supertree; d. the MRP supertree constructed by Clann (version 4.2.4). The inconsistent node and taxa were indicated by the red arrow and red frame respectively.
